# Alone, in the dark: The extraordinary neuroethology of the solitary blind mole rat

**DOI:** 10.7554/eLife.78295

**Published:** 2022-06-08

**Authors:** Yael Kashash, Grace Smarsh, Noga Zilkha, Yossi Yovel, Tali Kimchi

**Affiliations:** 1 Department of Brain Sciences, Weizmann Institute of Science Rehovot Israel; 2 School of Zoology, Faculty of Life Sciences, Tel Aviv University Tel Aviv Israel; Harvard Medical School United States; Harvard University United States

**Keywords:** blind mole rat, solitary, aggression, subterranean, neuroethology, social behavior

## Abstract

On the social scale, the blind mole rat (BMR; *Spalax ehrenbergi*) is an extreme. It is exceedingly solitary, territorial, and aggressive. BMRs reside underground, in self-excavated tunnels that they rarely leave. They possess specialized sensory systems for social communication and navigation, which allow them to cope with the harsh environmental conditions underground. This review aims to present the blind mole rat as an ideal, novel neuroethological model for studying aggressive and solitary behaviors. We discuss the BMR’s unique behavioral phenotype, particularly in the context of ‘anti-social’ behaviors, and review the available literature regarding its specialized sensory adaptations to the social and physical habitat. To date, the neurobiology of the blind mole rat remains mostly unknown and holds a promising avenue for scientific discovery. Unraveling the neural basis of the BMR’s behavior, in comparison to that of social rodents, can shed important light on the underlying mechanisms of psychiatric disorders in humans, in which similar behaviors are displayed.

## Introduction

**Figure 1.**
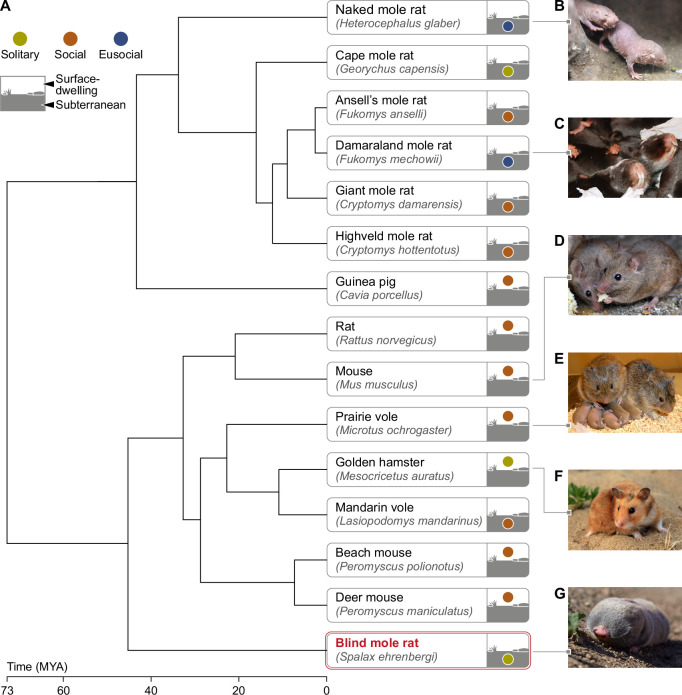
Phylogeny of mole rats and relatives subterranean and surface-dwelling rodents. (**A**) Phylogenetic tree generated by TimeTree software (http://www.timetree.org) displaying the evolutionary divergence of the BMR in relation to primary rodent species mentioned in this review. (**B**) Naked mole rat (*Heterocephalus glaber*) (**C**) Damaraland mole rat (*Fukomys damarensis*) (picture courtesy of Dr. Markus Zöttl) (**D**) Mouse (*Mus musculus*) (**E**) Prairie vole (*Microtus ochrogaster*) (picture courtesy of Prof. Larry Young) (**F**) Golden hamster (*Mesocricetus auratus*) (**G**) Blind mole rat (*Spalax ehrenbergi*). Time of divergence is indicated on the time scale, in millions of years ago (MYA). The color of the circle in the icon of each rodent indicates the sociality level (solitary/social/eusocial) of the species. The location of the circle distinguishes surface-dwelling from subterranean rodents. Subterranean rodents were defined as such, if they spend most of their lives in sealed underground burrows ( Nevo, 1999).

In the animal kingdom, social behavior strategies vary substantially within and between species, from a completely solitary lifestyle, to a eusocial way of life (reviewed by [Zilkha et al., 2021]). In social mammalian species, social structures of dyads, families or larger groups of various sizes are common (Prox and Farine, 2020), and social isolation is experienced as a potent stressor, which often leads to debilitating psychological, behavioral, and physiological effects (Mumtaz et al., 2018; Leigh-Hunt et al., 2017). In humans for example, prolonged social isolation due to the COVID-19 pandemic, was reported to have led to increases in cardiovascular disease cases, and cognitive deteriorations like depression and anxiety, in the worldwide population (Pietrabissa and Simpson, 2020; Taheri Zadeh et al., 2021). Social isolation in prairie voles was shown to produce cardiovascular dysfunctions and depression (McNeal et al., 2014), and socially isolated male mice showed impaired GABAergic function and increased aggressiveness (Matsumoto et al., 2005). In some species, however, social isolation is not experienced as a stressor, as they have evolved for solely solitary living. Within these species are large land predators such as the Eurasian lynx (*Lynx lynx*) (Breitenmoser-Würsten et al., 2007), and small rodents such as Heermann’s kangaroo rats (*Dipodomys heermanni*) (Yoerg, 1999). Interestingly, out of 19 known subterranean rodent genera, 14 are solitary (Nevo, 1979). Even more remarkable is that within this subterranean niche, mole rats range across the entire spectrum of sociality. For example, the naked mole-rat (NMR; *Heterocephalus glaber*) and Damaraland mole-rat (*Fukomys damarensis*) are eusocial, residing in large underground communal colonies, and are in fact the only two known eusocial species within the Mammalia class (Bennett and Jarvis, 1988; Barker et al., 2021a; Figure 1). Highveld mole-rats (*Cryptomys hottentotus*) are social, living in small, transient familial groups (Bennett and Faulkes, 2000; Moolman et al., 2006; Figure 1), and Cape mole-rats (*Georychus capensis*) live alone in solitary burrows (Du Toit et al., 1985; Figure 1). This variation of social strategies amongst subterranean rodent species provides a unique opportunity for comparative studies to investigate the neural and evolutionary substrates driving the transitions across the ‘social scale’ (Kimchi and Terkel, 2002; Nevo, 1999).

On this vast range of sociality among subterranean rodents, the blind mole rat (BMR), of the *Spalax ehrenbergi* superspecies (Family: Spalacidae), represents an extreme. It is an exceedingly solitary and territorial subterranean rodent that lives entirely within self-excavated underground tunnel systems. The BMR, weighing 100–250 g (Lacey et al., 2001), can be found across a variety of habitats in the middle east (Nevo, 1961; Nevo, 1985). Thus far, BMRs have taken the interest of scientists mostly due to their physiological adaptations to the hypoxic underground habitat, high longevity of 20 years or more, and cancer resistance (Manov et al., 2013; Schmidt et al., 2017; Shams et al., 2005). Here, we propose the BMR as a model for deciphering the mechanisms underlying anti-social, solitary behaviors, and for studying extreme aggressiveness manifested by individuals of both sexes. This model might be a crucial piece in the 'neural basis of social behavior' puzzle. Mice (*Mus musculus*) and rats (*Rattus norvegicus*), the most commonly used animal models in neuroscience (Ellenbroek and Youn, 2016), are considered highly social animals, and thus are not suitable for answering many scientific questions regarding the mechanisms controlling naturally-occurring anti-social and solitary behaviors. In recent years, there is a growing consensus among neuroscientists about the need for diversification of animal models (Brenowitz and Zakon, 2015; Hale, 2019; Remage-Healey et al., 2017; Yartsev, 2017). We suggest that the blind mole rat makes an ideal model organism for unraveling many neuroethological questions, such as: what is it in the BMR’s brain that makes it so aggressive and territorial? Why are social stimuli considered stressors for this animal? What are the sensory signals, circuits and neuromodulators controlling these behaviors? Are there evolutionary conserved mechanisms controlling anti-social behaviors across mammalian species? In addition, uncovering the brain mechanisms underlying the BMR’s sensory adaptations, its exceptionally solitary lifestyle and its extreme conspecific aggression, can serve as a prolific path for studies of brain functions associated with similar behaviors in humans. BMRs can serve as a model for understanding the neural circuits and mechanisms underlying congenital anti-social and aggressive behaviors. This may provide new knowledge on the neural basis of socially related psychiatric disorders in humans. For instance, people diagnosed on the autism spectrum, those who suffer from antisocial personality disorders, depression or neurodegenerative diseases like Alzheimer’s, often present a decrease in socialization and increased aggressiveness (Azevedo et al., 2020; Dutton and Karakanta, 2013; Gilley et al., 1997; Mazurek et al., 2013; Swearer et al., 1988).

**Figure 2.**
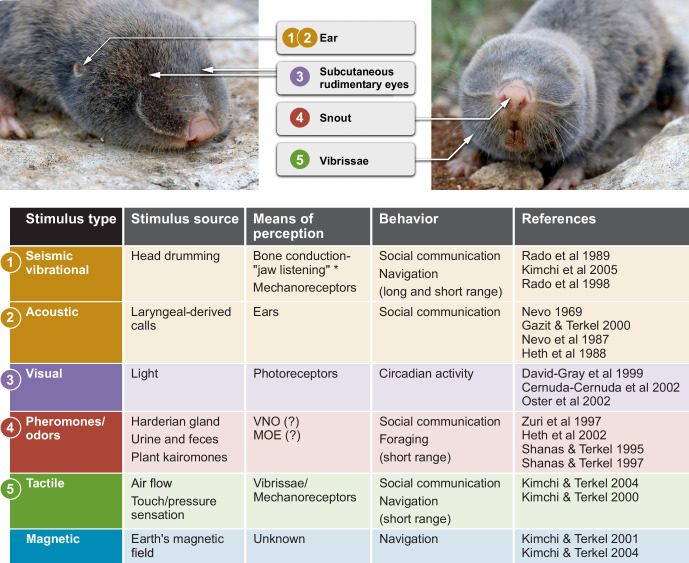
Sensory perception and unique adaptations in the blind mole rat. External morphology of the blind mole rat from a side (left) and front (right) view. Arrows point to key adaptive morphological features for sensory perception. Numbers indicate the corresponding sensory modality in the table below. The stimulus type, stimulus source, means of perception and type(s) of behavior are summarized. *Vibrational signals are perceived through the lower jaw and transmitted to the inner ear by means of bone conduction. Abbreviations: VNO, vomoronasal organ; MOE, main olfactory epithelium. Photo credit: Aviad Bar.

**Figure 3.**
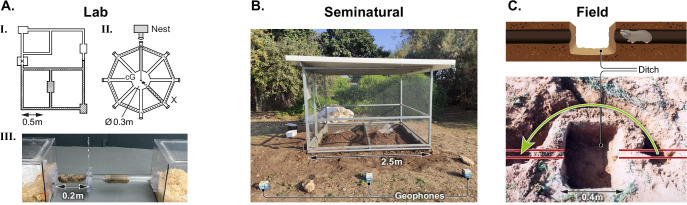
Experimental setups for the behavioral research of blind mole rats. (**A**) Laboratory experiments: I. Schematic representation of an experimental tunnel system made of Perspex tubes, used for the first successful breeding of BMRs in the lab. Tubes were arranged to simulate two territories with a connecting, dirt-filled nest box for mating. II. A wheel-shaped maze used for a navigation study of BMRs, consisting of eight tubes (routes) radiating from a central circle. III. An experimental setup for studying dyadic interactions between BMRs. A tube connects two Perspex home cages, with a perforated divider preventing physical contact of the two animals. (**B**) A seminatural setup: a metal mesh cage fully enclosed underground, with a central metal mesh divider allowing naturalistic experiments of two individuals simultaneously. (**C**) Field experiments: Schematic representation and photo of a field experiment demonstrating a bypass tunnel (green arrow) dug by a wild blind mole rat, following seismic ‘echolocating’ detection of a barrier. The barrier comprised of a ditch created by the researchers, marked in the illustration, which blocked the original tunnel (red lines).

In the course of this review, we explore the wealth of neuroethological opportunities in the BMR, relating to social behavior and interactions, foraging and navigation. We first describe the BMR’s extremely solitary behavior, its foraging and food-caching strategies, and the transient shift in sociality it needs to make with the onset of the breeding season. We also describe the BMR’s unique sensory adaptations to the underground habitat (Figure 2), including its remarkable demonstration of brain plasticity. The BMR’s magnetoreception ability is described, as well as its visual, auditory, somatosensory and olfactory systems. Yet, many questions regarding the neuroethology of the blind mole rat remain to be answered, preferably by combining lab, semi-natural and field studies (Figure 3). Here, we discuss some of those open questions, by summarizing what is known to date about blind mole rats and other closely related social and solitary mole rat species from a neuroethological point of view. This, we hope, will assist in gaining further insights on the neural basis of this extraordinary animal’s behavior, which we consider an excellent neuroethological model.

### Behavior of the blind mole rat

#### Territoriality and aggression

**Figure 4.**
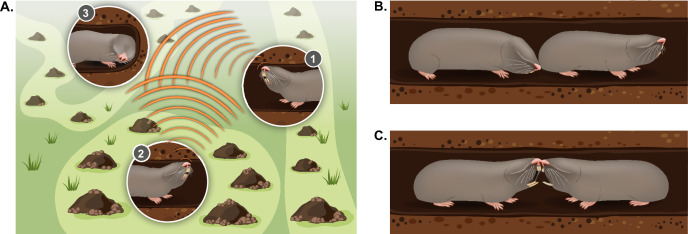
Typical social behaviors displayed by blind mole rats. (**A**) An illustration of the ’social communication network' between BMRs in neighboring territories using seismic signaling. BMRs #1 and #2 are head drumming back and forth on the tunnel ceiling; BMR #3 is ‘jaw listening’ by pressing its jaw against the tunnel wall. (**B**) Courtship behavior: a male BMR sniffing the genitals of a female BMR as part of the courtship process during the mating season. (**C**) An aggressive encounter between an intruder and a territory holder, including teeth baring and biting. Aggressive encounters typically lead to injuries and/or death of one or both rivals.

With the exception of short mating and lactating periods during the winter (the wet season), male and female blind mole rats reside alone throughout the year, patrolling their tunnels and defending their territories from conspecifics and congenerics (Nevo, 1961; Rado et al., 1993). For the BMR, close encounters from territory encroachment are experienced as extremely stressful, and usually result in severe physiological stress and extreme aggression, frequently leading to the death of one or both individuals (Nevo, 1961; Nevo, 2013; Zuri et al., 1998b, Zuri and Terkel, 1998). Agonistic and aggressive behaviors in BMRs are manifested by ‘retreat and attack’ postures and by ‘bulldozing’ a soil plug using the snout (Guttman et al., 1975). Other aggressive gestures include strong strikes and opponent-pushing with the head, open mouth sniffing (exposed incisors; Figure 4) and biting (Nevo et al., 1986). These behaviors were shown to increase where there is more competition for resource availability (Nevo et al., 1982). Females were reported to present lower aggression intensity compared to males (Nevo et al., 1986) nevertheless, the behavioral component of these behaviors was shown to be independent of testosterone levels in male BMRs (Gottreich et al., 2001). The territorial nature, extreme seasonality of breeding, and subterranean lifestyle of blind mole rats make them an excellent model for examining decision making, sensory perception in the dark and neural substrates underlying the transition from anti-social to social behaviors during the reproductive season.

Like other territorial animals, the spatial ecology and social organization of the blind mole rat can be considered a type of social communication network. In this network, repeated communication signals detected from a distance may advertise locations of neighbors and function to maintain boundaries and conspecific spacing (McGregor, 1997; Figure 4). Thus, the BMR’s specialized vibrational long-distance communication system, in the form of ‘head drumming’ (see section ‘Vibrational communication’), creates a specialized communication network between neighbors (Heth et al., 1987; Rado et al., 1987). This network is ever-shifting and dynamic. During the active winter months, individuals have many adjustments in territory size and location (Zuri and Terkel, 1996), and they must decide where to tunnel and whether to avoid or engage a neighbor in order to expand their territories. These decisions are likely heavily influenced by information from the head drums and by the individual’s experience. It has been noted that BMRs engage in head drumming ‘duets’ back and forth across territories (Rado et al., 1987), but the types of information encoded in the signal (for example the signaler’s level of dominance, sex, age, or individual identity) are not well understood (Randall, 2013; Seyfarth and Cheney, 2003; Tibbetts and Dale, 2007). Furthermore, whether additional signal features are modified in heightened motivational states (for example high vs. low aggression) has not been addressed (Bradbury and Vehrencamp, 2011). Understanding the neural processing underlying these finer cues of vibrational signaling dynamics, and how they result in a behavioral decision, will provide the mechanistic basis of territory maintenance and social avoidance, as well as social approach (i.e. finding a potential mate).

#### Reproductive behavior

BMRs are seasonal breeders (Shanas et al., 1995), for whom the active rainy season represents a crucial time in which males decide to burrow new tunnels to navigate toward a female. The approached female, on her end, can engage in either territorial battles or mating (Nevo, 1961; Nevo, 1969). Targeting and navigating underground toward a distant female is a complex sensory challenge, which is likely facilitated initially by long-distance head drumming, along with magnetic compass orientation (Kimchi and Terkel, 2001) and path integration for navigation (Kimchi et al., 2004). At a close distance to the female’s tunnel, courtship ensues, involving multimodal signaling including seismic head drums, vocal ‘purrs’ and olfactory cues, and engaging in tactile behaviors until the female decides to mate with the male (Gazit and Terkel, 2000; Nevo, 1961; Nevo, 1969; Shanas et al., 1995; Figures 2 and 4). Copulatory pairs spend brief periods together, retreating to their individual territories after mating (Gazit and Terkel, 2000).

A female will respond to the male’s courtship gestures based on her sensory perception and prior experience, to ultimately allow a typically aggressive male to approach, and subsequently mate with (Kavaliers and Choleris, 2017). To date, the mate choice strategy of female blind mole rats has not been thoroughly studied. One particular study examined reactions of females to calls from males belonging to the four chromosomal species of blind mole rats in Israel (2n=52; 2n=54; 2n=58; 2n=60) (Nevo et al., 1987). It was observed that estrous females are able to discriminate chromosomal subspecies of male BMRs by their courtship calls, and that in most cases, estrous females would prefer the calls of their homospecific mates over those of heterospecific ones (Nevo et al., 1987). Furthermore, the switch from extremely avoidant behaviors to actively seeking and encountering a mate represents a drastic shift in behavior by both males and females. Another interesting shift in the BMR’s behavior accrues in juveniles, which switch from socially interacting with their mother and siblings, to presenting an aggressive behavior toward them. Gestation period in BMRs is 34 days, after which the female delivers a litter of 3–4 pups on average (Gazit et al., 1996). Mother and pups communicate by vocalizations in the first stages of ontogeny, and gradually shift to seismic communication of head drumming duets (Rado et al., 1991b). Interestingly, Gazit and Terkel, 1998 reported sex differences in mother-pup interactions prior to a complete dispersal, as mother’s communication and aggression toward male pups was longer and more frequent compared to those toward female pups. The higher aggression toward male pups could be the motivating factor that drives juvenile males to disperse earlier than females, and establish more distant territories, preventing inbreeding. Overall, the transition from a social lifestyle during pup rearing, to a solitary lifestyle, is accompanied by increased aggression toward the pups, alongside increased aggression between siblings (Gazit and Terkel, 1998; Zuri and Terkel, 1998). This process leads to the gradual dispersal of the juveniles, starting approximately 2 months after parturition (Gazit and Terkel, 1998), and ending in the individual establishment of their own solitary territories at the age of approximately 3 months (Rado et al., 1991b).

The biological basis of these social lifestyle switches is unknown. Many environmental signals are known to act as timing cues, that alter the neuroendocrine substrates that regulating reproductive physiology and behavior (Follett, 2015). Despite residing almost exclusively underground, BMRs were shown to integrate photoperiodic changes and entrain a circadian activity rhythm by light received through their atrophied eyes (see section ‘Circadian rhythm’) therefore, changes in day length may be involved in the shift from solitary to mate-seeking behavior. In male prairie voles (*Microtus ochrogaster*), alteration of both temperature and photoperiod caused regression in synthesis of gonadotropin-releasing hormone, and in their reproductive function (Kriegsfeld et al., 2001). Thyroid hormones were also implicated in seasonality and breeding, as was demonstrated by Yoshimura and colleagues (reviewed in [Nakane and Yoshimura, 2014; Yoshimura, 2013]). Since BMRs breed in the wintertime, when ambient temperatures drop, it is possible that consequent elevated T3 levels enhance the onset of their reproductive behavior. Other environmental cues such as soil moisture content, sudden abundance of food associated with the rainy mediterranean winter, and even the composition of food resources, most probably play a role in the initiation of the breeding season. For example, in female townsend’s voles (*Microtus townsendii*), the addition of a specific plant metabolite during the non-breeding season, increased serum follicle-stimulating hormone levels and the size of reproductive organs (Schadler et al., 1988), and also resulted in a 4-week advancement of sexual maturation and breeding (Korn and Taitt, 1987).

The neural mechanism underlying behavioral plasticity in the ‘anti-social brain’ can be addressed within the framework of the 'Social Behavior Network', the set of interconnected brain regions implicated in the control of multiple forms of social behavior (Cummings and Ramsey, 2015; O’Connell and Hofmann, 2011; Prounis and Ophir, 2020).

#### Foraging behavior

BMRs are generalists that forage and cache encountered food items, such as geophytes and herbaceous plants (Galil, 1967; Heth, 1989, Lövy et al., 2015). The BMR’s foraging and food-caching behaviors are essential to ensure survival throughout the year (Rado et al., 1993; Vleck, 1979). When foraging in densely populated areas, BMRs must decide in which direction to tunnel based on environmental conditions such as moisture content, plant olfactory compounds and locations of nearby neighbors, from whom they must avoid (Heth et al., 2002; Rado et al., 1993; Gazit et al., 1997). Digging is very energetically costly. Thus, it likely benefits subterranean rodents to collect as many food types as they encounter within the habitat (Heth, 1989, Heth and Todrank, 2007, Nevo, 1961; Vleck, 1979).

In the summer, individuals stay largely inactive in tunnels at deeper and cooler depths (>40 cm) (Rado et al., 1993), where food is less available. Food caches support the BMR’s diet during this season (Lövy et al., 2015). In addition to remembering the location of their food caches, food-caching animals are often faced with the task of remembering which food items are stored, and the timing of storage, as different food items expire at different rates (Smith and Reichman, 1984). This “what, when, where” type of memory is referred to as episodic-like memory and has been well studied in birds (Roberts, 2006), but was also demonstrated in rats (Babb and Crystal, 2006; Yi et al., 2021). BMRs were shown to have far superior spatial memories compared to rats, learning a maze significantly faster, and retaining 45% of their performance 120 days later (Kimchi and Terkel, 2001). Other mole rat species, the Damaraland and Cape mole rats, have been shown to be able to learn and retain memory of the location of food rewards within a maze (Costanzo et al., 2009). While it is unclear how many food caches BMRs may have branching off their tunnels, which can be as long as 100 m, it is likely that their excellent spatial memory facilitates food cache revisiting (Lövy et al., 2015). Such high cognitive abilities probably integrate high spatial memory with specialized sensory abilities such as magnetoreception (Kimchi et al., 2004; Kimchi and Terkel, 2001), in food caching mole rat species (Lange et al., 2005). The underlying mechanisms, as well as basic behaviors involved in revisiting food caches and selecting food items, in line with prior life events (for example parturition), can be examined with both lab and semi-natural setups (Figure 3).

### Sensory adaptations to a subterranean, solitary lifestyle

#### Visual system

##### The BMR’s eye

While blind mole rats appear as if they have no eyes, they do possess rudimentary eyes (less than 1 mm in diameter), that are covered by furred skin (Cooper et al., 1993b, Haim et al., 1983; ; Rado et al., 1992; Figure 2). The lens of the BMR is degenerated and embedded within a hypertrophied harderian gland (Cooper et al., 1993b, Haim et al., 1983; Rado et al., 1992). The BMR’s retina contains < 900 ganglion cells (Cooper et al., 1993a), a significantly low number compared to >50,000 cells found in rats, for example (Gao et al., 2016). Moreover, brain structures involved in image processing are severely regressed and dysfunctional in the BMR (Cooper et al., 1993a). Multiple studies demonstrate that the non-functional visual centers in the BMR’s brain have been replaced by the auditory modality. For instance, the dorsal lateral geniculate body, which is the primary visual nuclei in the thalamus of sighted mammals, that sends visual information to the visual cortex, was reported to be strongly activated by auditory stimuli in BMRs (Bronchti et al., 2002; Bronchti et al., 1989; Heil et al., 1991). Although the BMR’s retina lacks short-wave opsine cones (David-Gray et al., 2002), it includes at least three types of photoreceptors (rodopsin, coneopsin and L/M cone opsin) that collect the scant light penetrating the soil (Burda, 2006; David-Gray et al., 1999; Esquiva et al., 2016). Overall, the BMR’s eye can be considered as a light-meter corresponding to the 'non-image forming' system found in the eye of sighed mammals.

##### Circadian rhythm

In mammals, light perceived through the retina uses the common photoreceptors (i.e. rods and cones) for image formation, and the photoreceptor melanopsin for the non-image forming system. Melanopsin is the driving force for the circadian timing system, that initiates the animal’s physiology and behavior in distinct times and periods (Freedman et al., 1999; Hannibal, 2021). The BMR’s eyes respond to light stimulation and provide information used to entrain circadian and annual cycles generated by the suprachiasmatic nucleus (SCN), through the neuronal pathway of the retinohypothalamic tract (Cernuda-Cernuda et al., 2002; David-Gray et al., 1999; Hannibal, 2002; Moore et al., 1995; Oster et al., 2002). Therefore, despite being blind, light induces gene expression in the SCN (Vuillez et al., 1994) and entrains circadian rhythms of locomotor behavior in the BMR (David-Gray et al., 1998; Rado et al., 1991a). These reactions to light rely upon retinal photoreceptors, since enucleation abolished them (David-Gray et al., 1998; Pevet et al., 1984; Rado et al., 1991a). In addition to the atrophied eyes, the BMR’s harderian gland is also involved in photoperiodic perception, as it is able to synthesize melatonin (Balemans et al., 1980), and it was shown to be involved in photoperiodic detection (Pevet et al., 1984). Melatonin receptors were also found in the BMR’s harderian gland, and were suggested to take part in a negative feedback control of melatonin production (Gilad et al., 1997). Interestingly, the eusocial, subterranean Damaraland mole-rat was also shown to possess a circadian melatonin rhythm that is modulated according to photoperiodic changes (Richter et al., 2006).

Insights on light perception in the BMR emphasize its relevance as a model for studying re-routing of sensory modalities into processing areas of other sensory modalities, after evolution has neutralized its original output. Such cross-modality reorganizations in animal models are often induced artificially (for example by sensory deprivation or neuronal manipulation). However, the visual system of the blind mole rat is naturally and congenitally adapted to life in the dark, rendering it a very attractive subject for studying evolutionary plasticity in the central nervous system (CNS) and for revealing the mechanism of neuronal compensation.

### Magnetoreception

Magnetoreception, the ability to perceive the earth’s magnetic field, is a sensory modality observed in various vertebrate species (Begall et al., 2014), some invertebrates taxa (Hsu and Li, 1994; Lee et al., 2021) and even in plants (Galland and Pazur, 2005). Unlike surface-dwelling rodents, subterranean rodents lack some external stimuli that are normally used for spatial orientation, such as stars, sun light azimuth, and other visual cues. Kimchi and Terkel, 2001 demonstrated that blind mole rats have magnetoreception abilities, and that they rely on them when choosing the locations for their nests and food caches. When shifting the polarity of the earth’s magnetic field by 180̊, BMRs shifted the location of their preferred nest and food cache sites accordingly (Kimchi and Terkel, 2001). To this day, there is a debate regarding the magnetosensory organ of terrestrial animals, and there is evidence for both light-dependent and light-independent mechanisms of magnetoreception. Are magnetoreceptors located in the minute eyes of mole rats, or are they located elsewhere? In birds and other vertebrates, the ability to use the earth’s magnetic field for spatial orientation was shown to be light dependent, apparently perceived through eye receptors (Phillips and Borland, 1992; Wiltschko et al., 2008; Wiltschko et al., 1993; Wiltschko and Wiltschko, 1982). In addition, following surgical removal of their eyes, individual Ansell’s mole-rat (*Fukomys anselli*) demonstrated impaired magnetic sensing, supporting the hypothesis that in mole rats, magnetoreception receptors are located in the cornea (Caspar et al., 2020). However, some observations in BMRs suggest that the their magnetoreception is independent of light, as experiments were conducted in total darkness (Kimchi and Terkel, 2001). This finding is supported by a study of another mole rat species, the Zambian mole rat (*Fukomys amatus*), that was shown to be independent of light in its perception of the earth’s magnetic field (Marhold et al., 1997). Further research based on magnetic compass orientation in mole rats, and in particular the blind mole rat, is needed, as is further research investigating the location of relevant magnetoreceptors and sensory pathways.

### Auditory system

#### Hearing sensitivity

For any animal that is active where and when visual cues are scarce, acoustic vocalizations usually become an important sensory modality for navigation (for example echolocation), or for detection and communication with conspecifics (Bradbury and Vehrencamp, 2011). However, for subterranean mammals including blind mole rats, the underground environment creates very specific acoustic conditions. Tunnels create large surface areas at close proximity to the emitter of the signal, causing high levels of attenuation of high frequencies sounds through absorption, and simultaneously amplification of noise from reverberation (Begall et al., 2007). Accordingly, fossorial and subterranean animals generally hear better at low frequencies and have restricted hearing ranges, compared to surface-dwelling rodents that have been known to produce frequencies as high as in the ultrasonic range (Dent et al., 2018; He et al., 2021). The Highveld mole rat (Müller and Burda, 1989) and the Zambian mole rat (Brückmann and Burda, 1997), both subterranean rodents, are more sensitive to very low frequencies (0.8 kHz) while the surface-dwelling feral house mouse (Heffner and Masterton, 1980) is most sensitive to higher frequencies (16 kHz).

In addition, peak sensitivities for hearing vary substantially among rodents (Gerhardt et al., 2017; Heffner and Heffner, 1990; Heffner and Heffner, 1992; Heffner and Heffner, 1993). For example, peak sensitivity in the surface-dwelling guinea pig (*Cavia porcellus*) is –9 dB SPL (sound pressure level) (Prosen et al., 1978), while in the subterranean naked mole rat it is just 35 dB SPL (Heffner and Heffner, 1993). Results from behavioral audiograms showed that trained blind mole rats can hear between 54 Hz and 5.6 kHz at an amplitute of 60 dB SPL , with peak sensitivity at 1 kHz at 32 dB SPL (Heffner and Heffner, 1992). Additionally, blind mole rats have poor sound localization abilities, as demonstrated from playback of left and right speakers with 180^o^ of separation (Heffner and Heffner, 1992). Blind mole rats cannot localize signals less than 400 ms in duration, and performed best at localizing sound bursts of 1.2ms (Heffner and Heffner, 1992). The lower hearing sensitivity and minimal localization ability are apparent in the morphology of the BMR, which lacks an external pinna of the outer ear (see ear opening location in Figure 2). The BMR’s middle and inner ear are uniquely structured, creating inefficient transmission of airborne sound (Rado et al., 1989).

#### Vocalizations

The low-frequency repertoires of different mole rats vary across species, consisting of a variety of social calls (Knotková et al., 2009). Giant mole rats (*Fukomys mechowii*) were demonstrated to have a repertoire of 14 call types as determined by behavioral context, such as teeth-fencing and predator alarm, and by spectro-temporal features (Bednářová et al., 2013). In accordance with their low frequency hearing ranges, these calls are low in frequency, with peak frequencies of less than 5 kHz (Bednářová et al., 2013). The eusocial naked mole rat was recently shown to have dialects in the ‘soft chirp’ call type across colonies that signifies group membership (Barker et al., 2021b). Since BMRs are spatially isolated it is unlikely that their social calls can be transmitted or detected across territories through the thick soil substrate (Heth et al., 1986; Narins et al., 1992).

The full vocal repertoire of the BMR has not yet been described. At least six call types determined by behavioral context, all with frequencies below 8 kHz, have been noted thus far (Capranica et al., 1974). Different call types are used to facilitate mother and pup communication (Capranica et al., 1974; Gazit and Terkel, 1998; Rado et al., 1991b), and used in male courtship displays during the mating season (Gazit and Terkel, 2000; Nevo, 1969). These courtship calls differ in frequency and temporal components across the *Spalax* chromosomal subspecies, and appear to play a role in assortative mating between the neighboring populations (Heth et al., 1988; Nevo et al., 1987) (see section ‘Reproductive behavior’). Distress calls with peak frequencies between 4 and 6 kHz have also been opportunistically observed in lab studies (Bronchti et al., 1989). However, variation in spectro-temporal features by individuals and by level of stress, as well as variation in use in the context of multimodal signaling (for example head drums and physical encounters) have not been studied.

#### Vibrational communication

The auditory system of the BMR is uniquely adapted to detect and process non-vocal vibrational (seismic) signals (Figure 4). The use of substrate-borne vibrational signals in animals is not well understood, but has been observed in elephants, arthropods such as spiders, some reptiles, and a number of fossorial or semi-fossorial rodents, including kangaroo rats (Heteromyidae), African mole rats (Bathyergidae), and blind mole rats (Spalacidae) (Hill, 2001; Narins et al., 2009). The mechanism for production of vibrational signals varies across taxa and species. These signals are used for communication between conspecifics, for mating and/or inter-individual spacing (for example territory maintenance), targeting prey and even for detecting obstacles (Hrouzková et al., 2018; Kimchi et al., 2005; Narins et al., 2009; Narins et al., 1997; Randall and Lewis, 1997).

A BMR produces vibrational signals by drumming on the ceiling of its tunnel with the large, flattened portion of its head, producing bursts of head drums (approx. 2–4 drums per burst, 0.3 s duration) (Heth et al., 1987; Rado et al., 1987; Figure 4). Seismic signals range in the frequency of approximately 100–250 Hz and may travel as far as 10 m or more (Heth et al., 1987; Rado et al., 1987). Perception of seismic signals is achieved in two ways, one by pressing the side of their lower jaw against the tunnel wall, which transmits the signals to the inner ear by means of bone conduction (Mason et al., 2010; Rado et al., 1989; Figure 4A). The Second is via mechanoreceptors located on their paws (Kimchi et al., 2005). "Jaw listening” behavior was observed in laboratory conditions in which BMRs in Perspex tubes alternated between head drumming and placing their jaws on the tube wall (Heth et al., 1987; Rado et al., 1987). In field experiments, while radio-tracking BMRs using very high frequency telemetry, Zuri & Terkel observed that individuals patrolled their territories with regular pauses for 5–120 sec, suggesting that they stop to ‘listen’ for seismic activity of neighbors (Zuri and Terkel, 1998). In addition, in a lab study using a maze, individuals paused at maze junctions and placed their jaws against the tube (Kimchi and Terkel, 2004). Providing a vibration stimulus in contact with the lower jaw evoked responses in the auditory brainstem, and bilaterally deafened mole rats, showed loss of response and eventual ceasing of head drumming behavior in the course of 4–6 weeks (Rado et al., 1998). Furthermore, the occipital cortex, usually associated with vision, was shown to be activated by frequencies corresponding to both vibrational and acoustic signals (Bronchti et al., 2002; Heil et al., 1991; Sadka and Wollberg, 2004). In one study, cortical activation was demonstrated via playbacks of acoustic stimuli including clicks, noise, and tones while collecting extracellular single unit recordings in the cortex (Sadka and Wollberg, 2004). Specifically, two clusters of cells fired in response to frequencies centered around 2.5–4.4 kHz, matching airborne social vocalizations, and to 100 Hz, matching the frequency of long-distance vibratory signals (Sadka and Wollberg, 2004). Further experiments using vibrational stimuli and neural recordings or MRI may confirm the convergence of vibration and auditory stimuli processing in the BMR cortex.

The general understanding of the behavioral discrimination and sensitivity of perception of vibrational waves in the subterranean environment is greatly lacking (Hill, 2009; Mortimer, 2017). The sensory sensitivity to fine signal differences and its encoding in the cortex would be a great advancement in understanding of the seismic sensory system of the BMR.

### Somatosensory system

In addition to the auditory cortex, it was further shown that the BMR’s somatosensory cortex also extends far into the occipital cortex (Necker et al., 1992; Rehkämper et al., 1994). This finding is a clear example of the evolutionary plasticity of the BMR’s brain. The somatosensory system in the isocortex of the blind mole rat was shown to be 1.7 times larger compared to the laboratory rat, when taking into consideration the differences in body weight and brain size of the two species (Mann et al., 1997). Subterranean rodents, including BMRs, have a highly developed tactile sense, used as a mechanism of navigation in their complex tunnel system, and as a mechanism for detecting substrate-borne vibrational signals (Necker et al., 1992; Nevo et al., 1991; Rehkämper et al., 1994). They possess sensory vibrissal hairs connected to sensitive mechanoreceptors dispersed over different body parts, contributing to efficient orientation and likely path integration in their narrow burrows (Bennett and Faulkes, 2000; Figure 2). Klauer and colleagues found that the dermal papillae of the rhinarium in the BMR contains a few Merkel cell-axon complexes and simple Meissner’s corpuscles. This might indicate the sensitivity of the rhinarium to vibrations and its involvement in perception of seismic signals in terms of rhythm, amplitude and frequency (Klauer et al., 1997). Nevertheless, it is worth noting that similar receptors were also found in the noses of rats (Silverman et al., 1986), a rodent species that is not known to use seismic communication. During maze learning and exploration tasks, BMRs consistently engage in a ‘wall seeking’ behavior, in which they press their bodies against the side of the wall, sometimes alternating sides, and brushing the vibrissae against the maze surfaces (Kimchi et al., 2004). When comparing maze learning and performance, blind mole rats performed significantly better in a narrow maze which allowed constant contact with the wall, compared to a maze twice the width of their body diameter (Kimchi and Terkel, 2000). In contrast, rats performed better in the wider maze (Kimchi et al., 2004). In nature, BMRs dig their tunnels to exactly the diameter of their own body width, in order to obtain maximal tactile information (Kimchi and Terkel, 2002). The vibrissae hairs may provide crucial information that assists the blind mole rats to rapidly orient and move through their tunnel. This further implies that sensory acquisition from hairs that are touching tunnel surfaces or receiving fine air movements is rapidly processed by somatosensory and motor circuitries. Additionally, for naked mole rats which use their teeth for digging, foraging, social encounters, and moving pups, the incisors are represented in approximately 31% of the somatosensory cortex (Catania and Remple, 2002; Park et al., 2007). We may expect to find similarly high representation of teeth in the BMR, which are also reliant on teeth for digging, foraging, and aggressive encounters.

In mammals, oscillating signals passed to the animal by direct contact with a solid substrate can be perceived by both auditory and somatosensory mechanisms. The role of the auditory system in detection of seismic signals was discussed earlier (in the 'Vibrational communication' section). Blind mole rats have been suggested to detect vibrational signals through the somatosensory system in addition to the auditory system. Kimchi and colleagues demonstrated that BMRs use seismic signaling as an echolocation behavior (Kimchi et al., 2005). In field experiments, blind mole rats were able to detect obstacles placed by the researchers in their tunnels (various size ditches), and repeatedly dug around or over them. The bypass strategy varied depending on the size of the obstacle (large/small ditches), likely to minimize energetic costs (Kimchi and Terkel, 2003a, Kimchi and Terkel, 2003b; Figure 3C). The BMRs in these controlled field experiments were hypothesized to head drum and detect the signals reflected from the substrate barrier (air), sensing the location and size of the barrier. Unlike head drums used for intraspecific communication, these drums were higher in frequency (250–300 Hz) and produced as a single hit rather than bursts at intervals of approximately 8 s (Kimchi et al., 2005). Through modeling, it was demonstrated that the amplitude and frequency of the wavelengths recorded by geophones are sufficient for rapid detection of reflected waves when the individual is 30–50 cm away from an obstacle (Kimchi et al., 2005). However, because the reflected waves were estimated to return to the individual faster than the individual could place its jaw against the tunnel (for 'jaw listening'), the research team suspected an alternative form of vibration detection. Experimentally, it was shown that BMRs can detect seismic signals produced by a mechanical shaker or a stimulus mole rat, also via their paws (Kimchi et al., 2005). The glabrous (hairless) skin of the front and hind feet of the BMR contains 15–20 lamellated Pacinian corpuscles, a type of mechanoreceptor known to be sensitive to high-frequency vibrations from 10 to 400 Hz in mammals (Kimchi et al., 2005; McIntyre, 1980). Currently, these corpuscles are the only proposed receptors for somatosensory seismic sensitivity used in echolocation in BMRs. These findings open a promising research avenue, examining the role of the somatosensory cortex in encoding seismic signals for orientation skills. We may expect a pattern of frequency, amplitude and temporal entrainment of cortical neurons for object discrimination (Prsa et al., 2021; Prsa et al., 2019). It is most likely that both the auditory and the somatosensory systems are involved in vibration processing, as is the case in humans and other animal species (Budinger et al., 2006; Campi et al., 2010; Roy et al., 2017).

### Olfaction

Olfaction, the sense of smell, plays an important role in detection of food, identification of conspecific territories and recognition of mates. For an animal as aggressive and territorial as the blind mole rat, which is also visually blind, the ability to identify odors from the surroundings is highly important in order to avoid intraspecific aggressive encounters. BMRs possess dorsoventrally flattened, wide, snouts (~15 mm wide in adults; personal observations) (Figure 2), that assist them in pushing out the excavated soil while burrowing, using bulldozing movements of their heads, consequently creating multiple mounds above the ground (Figure 4). BMRs have been shown to use olfaction to discriminate poisonous versus edible food items from the plant growth hormones, kairomones located within soil where plants had been growing (Heth et al., 1992, Heth et al., 2002).

The mammalian main olfactory system detects mainly environmental odorants (Sanchez-Andrade and Kendrick, 2009), while the vomeoronasal organ (VNO) located at the base of the nasal cavity, mainly detects intraspecific pheromones (Keverne, 1999). In mammals, particularly rodents, the VNO plays a major role in mediating socio-sexual behaviors (Cross et al., 2020, Halpern and Martınez-Marcos, 2003; Kelliher, 2007). As in rats (Addison and Rademaker, 1927), the VNO of the blind mole rat opens directly to the nasal cavity by a narrow canal, and is not connected to the oral cavity (Zuri et al., 1998a). In developing mole rats from infancy to adulthood, there is an approximately 8.5-fold increase in VNO length (from 1.5 mm at 4 days to 13 mm in adults) (Zuri et al., 1998a). This increase is much more substantial when compared to findings in African mole rat species (family *Bathyergidae*), which show postnatal vomeronasal epithelium (VNE) length increase of less than one-fold (Dennis et al., 2020). Moreover, in the naked mole rat, no postnatal volumetric growth was found in the VNE (Smith et al., 2007). This data might suggest that blind mole rats possess higher abilities for pheromone detection, in comparison to other mole rat species.

Most terrestrial vertebrates carry two large families of vomeronasal pheromone receptors: vomeronasal receptors type 1 (V1Rs) and type 2 (V2Rs) (Nei et al., 2008). While V1Rs are mainly involved in detecting air-borne molecules scattered in the air, V2Rs are commonly responsible for binding to water-soluble peptides abundant in aquatic environments (Shi and Zhang, 2007). Recently, it was shown that there has been a convergent reduction of V1Rs in different subterranean rodents, including the BMR (Jiao et al., 2019; Zhao, 2021), suggesting that pheromonal olfaction mediated by V1Rs might be reduced, as it may not be as useful as seismic communication in the underground tunnel environment.

Pheromonal signaling in the blind mole rat is used in short-distance social communication (Figure 4C). In the BMR’s underground tunnels, there is very restricted airflow, limiting the detection distance of the signal. The BMR secretes pheromones from the large harderian gland located at the ocular orbit by self-grooming (Shanas and Terkel, 1995). The harderian gland is sexually dimorphic in lipid and volatile composition, and in size and weight (larger in males) (Shanas and Terkel, 1999). Gland size and weight also correlate with total body mass and season of the year (Shanas and Terkel, 1999). Secretion of pheromones by grooming during social interactions is thought to signal submissiveness and appeasement (Shanas and Terkel, 1997).

Urine and feces are other prominent sources for odor stimuli used by many mammalian species in a variety of behaviors (Bronson, 1979; Doty, 1986; Macdonald et al., 1990). Male BMRs use urine and feces for scent marking, to proclaim their territories and prevent intrusion by competitors (Gazit et al., 1997). However, mole rat odors may also serve as sexual attractants. Heth and colleagues showed that specific pheromones in urine are important for both courtship and territoriality in males and females (Heth et al., 1992). A later study showed that female BMRs were attracted to males with high urinary testosterone levels, suggesting that testosterone and androstenedione behave as pheromones and have an olfactory effect (Gottreich et al., 2000). Follow-up studies showed that male BMRs kept in a lab facility have significantly lower androgens, testosterone and androstenedione levels compared to male BMRs in the wild, which might explain their low courtship behavior in the lab and the low breeding success rate in captivity (Shanas et al., 1995). Artificially induced estrous female mole rats were shown to be attracted to, and prefer male odors belonging to their chromosomal subspecies when auditory and tactile cues were excluded (Nevo et al., 1976). These data support the assumption that pheromones are highly involved in reproductive behavior and genetic speciation of the BMR. The mechanism allowing the mole rat to detects and process pheromone signals is yet to be determined.

### Neuromodulation of aggression and social behavior in subterranean mole rats

Various neuromodulators were shown to be involved in aggressive behavior and sociability in mammalian species in general, and in rodent species in particular (Insel and Young, 2000; Lee and Beery, 2019). In this section, we focus on oxytocin (OT), arginine vasopressin (AVP) and corticotrophin releasing factor (CRF) as three potential hypothalamic neurotransmitters that might be involved in regulation of the unique social behaviors of the BMR. Since very little is known regarding the neurobiology of the BMR, we expended this section to review the accumulating knowledge of other mole rat species, including the eusocial naked and African mole rats, and the solitary Cape mole rat.

#### Oxytocin and vasopressin

The neuropeptides oxytocin and vasopressin are strongly associated with various social behaviors such as social memory and attachment, sexual and maternal behavior, affiliation and aggression in humans and many other mammalian species (Anacker and Beery, 2013; Kompier et al., 2019; Lim and Young, 2006; Veenema and Neumann, 2008). These centrally released neuromodulators regulate neural activity in multiple brain regions involved in social behavior. In the eusocial NMR (Rosen et al., 2008) and African Ansell’s mole rat (Valesky et al., 2012), the majority of OT cells was found in the paraventricular nucleus (PVN) and supraoptic nuclei (SON), similar to many other mammalian species (Buijs, 1978; Dubois-Dauphin et al., 1989; Hermes et al., 1988; Sofroniew et al., 1979). Sex differences in the number of OT and AVP neurons were examined in several rodent species and were demonstrated in house mice, Brandt’s voles (*Lasiopodomys brandtii*), mandarin voles (*Lasiopodomys mandarinus*) (Qiao et al., 2013) and greater long-tailed hamsters (*Tscherskia triton*) (Häussler et al., 1990; Xu et al., 2010). However, no sexual dimorphism in OT neuron number was found in prairie voles (Yamamoto et al., 2004), Ansell’s mole rats (Valesky et al., 2012), and naked mole rats (Mooney and Holmes, 2013).

In the blind mole rat, no data was published thus far regarding the hypothalamic population and distribution of OT neurons. Preliminary data recently established in a comparative study in our lab, found that when considering the differences in brain size, the BMR’s PVN contains significantly lower numbers of OT neurons compared to mice. Furthermore, we found no indication of sex differences in the number of OT cells in the PVN of male and female BMRs (unpublished data). Moreover, while it is often assumed that male BMRs are more aggressive than females, our observations suggest similar levels of aggression in both sexes (unpublished data), which might be related to the sex similarities in OT distribution.

There are many evidence that link social cues with the number of OT and AVP cells in the hypothalamus. The presence of a social stimulus was shown to activate the OT system to promote adaptive social behavior responses; a study of the eusocial NMR that examined the effect of social status on the number of OT neurons, revealed that subordinate individuals have more OT-producing neurons in the PVN compared to breeding individuals (Mooney and Holmes, 2013). Peripheral administration of OT to the eusocial naked mole rat enhanced social behaviors such as in-colony huddling, time spent in close proximity to a conspecific and the number of its investigations, and this enhancement was blocked by co-administration of an OT antagonist (Mooney et al., 2014). This further strengthens the apparent role of oxytocin in the NMR’s social behavior. As for the African Ansell’s mole rat, no significant differences in OT and AVP neurons were found in individuals from different social and reproductive ranks (Valesky et al., 2012).

Oxytocin and vasopressin have been suggested to play a role in the regulation of agonistic, anti-social and aggressive behaviors in various rodents (Tan et al., 2019), and were implicated in the manifestation of autism spectrum disorders (ASD). For example, it was recently shown that *Shank3b* KO mice, a common mouse model of ASD, which present abnormal social behaviors, have reduced numbers of OT neurons in the PVN (Resendez et al., 2020). While most studies indicate that OT acts as a prosocial agent, reducing aggressiveness (Calcagnoli et al., 2014; Harmon et al., 2002; Takayanagi et al., 2005; Tsuda et al., 2011; Veenema et al., 2007; Winslow et al., 2000), some studies indicate that OT also enhances agonistic and aggressive behaviors (reviewed by [Beery, 2015]), mostly maternal aggression (Bosch et al., 2005; Oliveira et al., 2021, DeVries et al., 1997; Ferris et al., 1992), but also paternal aggression (Shabalova et al., 2020). For AVP, the majority of studies indicate that it is involved in the upregulation of aggressive behavior in rodents. For example, enhanced aggression due to early life stress (ES) was shown to be associated with upregulation of AVP expression in the PVN and SON of male rats (Veenema et al., 2006; Veenema and Neumann, 2009). On the contrary, mice (Tsuda et al., 2011) and mandarin voles (Wei et al., 2013) presented decreased aggression after ES, which was accompanied by a decrease in AVP neurons in the PVN. These findings indicate that AVP is a neuromodulator involved in the manifestation of aggressive behavior in rodents. The response of OT and AVP neuronal populations to social stimuli in the blind mole rat was not documented. As far as we know, no attempts were made to administer OT or AVP to BMRs, either centrally or peripherally. In addition to OT and AVP, important findings can arise from exploring their associated receptors in the solitary BMRs, as there are growing evidence for diversity in their genetic regulation, that might trigger variations in social behaviors (Donaldson and Young, 2008; Insel et al., 1991). For example, a genetic diversity in the levels of brain AVP receptor in monogamous prairie voles was demonstrated to influence sexual fidelity level in males (Okhovat et al., 2015). Expression levels of OT and AVP, both centrally and peripherally, have never been documented in BMRs, and could shed light on the basis of different social behaviors in males and females (for example [Bendesky et al., 2017; Yohn et al., 2017]). Taking into account findings from other rodents and other mole rat species in particular, we assume that OT and AVP neuronal populations play a key role in regulating social behaviors including aggression and agonistic phenotypes, in the BMR.

#### Corticotrophin-releasing factor

In the mammalian brain, CRF is most abundant in the PVN, central amygdala (CeA) and hindbrain (Merchenthaler et al., 1982). With initiation of the stress response, CRF neurons in the PVN are evoked to release CRF peptide at the median eminence, triggering adrenocorticotropic hormone release from the pituitary, resulting in elevated corticosteroid production from adrenal cells, and the manifestation of different survival behaviors (Jiang et al., 2019; Koob et al., 1994). In the CNS, CRF and its related peptides- urocortin I-III were shown to be involved in the regulation of social behaviors such as parental care, affiliation and aggression (Hostetler and Ryabinin, 2013).

The effects of CRF and its receptors in regulation of male aggression in rodent species depend on the site of its administration, dosage and experimental paradigm. In some studies, CRF administration, or elimination of one of its ligands or receptors (by a genetic knock-out), was shown to facilitate or decrease aggressive behavior (Breu et al., 2012; Coste et al., 2006; Elkabir et al., 1990; Mele et al., 1987), while in others, such manipulations had no effect (Gammie et al., 2005; Gammie and Stevenson, 2006).

In a comparative study, Coen found considerable differences in CRF abundance and receptor binding in the telencephalon, between the eusocial NMR and the solitary Cape mole rat (Coen et al., 2015). These differences might reflect the opposite social strategies of the two mole rat species.

Further research into the role of the OT, AVP, and CRF systems in sociability of the solitary BMR, and their comparison to findings in the eusocial NMR, could potentially add to our understanding of the neuromodulators and circuits governing anti-social behavior.

### Summary

In his review from 1991, Heiligenberg, 1991 discussed the different approaches used by scientists in the study of the neural basis of animal behavior, and described how often times, neuroethologists choose ‘champion species’ for their studies. These ‘champion species’ possess unique aspects of sensory or motor performance, that are linked to specialized neuronal structures in the CNS (Heiligenberg, 1991). Here we argue that the blind mole rat qualifies as a champion species in terms of its sensory performance, behavioral repertoire and special adaptations to underground life, which manifest in remarkable brain plasticity.

Being congenitally blind, *Spalax ehrenbergi* is deprived of visual inputs, and thus compensates via the diversion of the visual modality to the auditory modality. Cross-modal neuroplasticity in animal models is induced, in most cases, by experimental procedures (Izraeli et al., 2002; Rauschecker and Kniepert, 1994; Toldi et al., 1994a; Toldi et al., 1994b; Yaka et al., 2000). By using the blind mole rat as a model animal, we take advantage of its natural and congenital sensory deficit- its blindness.

Neuroscience and social behavior research has been, and still is, mostly conducted using highly social animal models, most commonly mice and rats (for example [Ellenbroek and Youn, 2016; Haney et al., 1989; Karamihalev et al., 2020; Scott et al., 2015; Shemesh et al., 2013]). Neuroethological studies have also been conducted on other rodent species, such as golden hamsters (Bunnell et al., 1970), prairie voles (McGraw and Young, 2010), gerbils (Martínez et al., 2019) and in the subterranean niche, the naked mole rat is a relatively common subject for research, being eusocial and easy to breed in captivity (Buffenstein et al., 2021). However, the blind mole rat is territorial and aggressive, and is extremely difficult to breed in the lab. It has been shown once that under careful conditions, the BMR can be bred in captivity (Gazit et al., 1996; Figure 3), nevertheless, as of yet no major breeding programs have been initiated. To our knowledge, there is no known way to determine the age of an adult BMR captured in the field, which adds to the uncontrolled variables involved in BMR research. Still, *Spalacinae* are very common in some regions, and research has been conducted successfully on wild-caught individuals (for example Gazit and Terkel, 1998; Gazit and Terkel, 2000; Kimchi et al., 2004; Kimchi and Terkel, 2001; Nevo et al., 1975; Figure 3A). Furthermore, no genetic manipulations or selective breeding are done on BMRs, and thus all naturally occurring properties and genetically determined mechanisms are conserved in this non-model organism. This is in contrast to laboratory mice and rats, which are subjected to generations of domestication and inbreeding, thus accumulating numerous mutations and consequently present many atypical features compared to wild populations in nature. Many natural behaviors of BMRs can and have been addressed in lab and semi-natural behavioral experimental setups related to the sensory systems outlined above (for example Zenuto et al., 2001; Figure 3). However, as in mice and rats, it is best to complement lab studies with natural experiments from a behavioral ecology approach, to collect the full suite of complex behaviors in the BMR.

In conclusion, a handful of animal models for the study of diverse biology and neurobiology is not sufficient. We have recently discussed the main biases in social behavioral research, and showed that science in general, and behavioral and neurobiological studies in particular, tend to rely mostly on male mice in a small confined laboratory environment (Zilkha et al., 2016). However, in order to understand the neural mechanisms underlying different types of social behaviors, non-traditional model organisms must be considered. The blind mole rat provides an excellent non-traditional model, whose neuroethology might also shed light on the neurobiological basis of social deficits in humans. Moreover, BMRs provides an almost untouched territory in the neuroscience field, rendering them a fascinating and exciting model organism for neuroethological research. Nevertheless, in order to utilize the unique features of BMRs in our studies and establish it as a new model in social neurosciences, some basic genetic, molecular and neurological data needs to be collected. A *Spalax ehrenbergi* genomic assembly has been deposited for a single female BMR (Fang et al., 2014). In addition, transcriptomic sequencing analyses have uncovered differential expression profiles in BMRs under different conditions related to underground stressors (Fang et al., 2014; Malik et al., 2011), and scRNA sequencing of microglia revealed similarities of BMRs to other rodents (Geirsdottir et al., 2019). An additional step forward would be mapping of the BMR’s brain morphology and functional circuits. This can be done by detailed histological analyses, accompanied by MRI. Furthermore, making commonly used viral tools for neural manipulations applicable for BMRs will assist in mapping the cascades underlying brain coding of social information. Recently, Hadi and colleagues demonstrated a promising breakthrough in the field of genetic engineering in mole rats, reporting a successful transformation of NMR cells, using a novel CRISPR gRNA library and lentiviral vectors (Hadi, 2020a; Hadi et al., 2020b). This is a major step forward in advancing the genetic tools used on subterranean rodents, and making them much more available for the research of mole rats in general, and the blind mole rat in particular.
